# Supplementary figures and images for: Real-Time Navigation in Google Street View® Using a Motor Imagery-Based BCI
Source: Sensors (Basel). 2023 Feb 3;23(3):1704. doi: 10.3390/s23031704 (PMC9921617; doi:10.3390/s23031704)

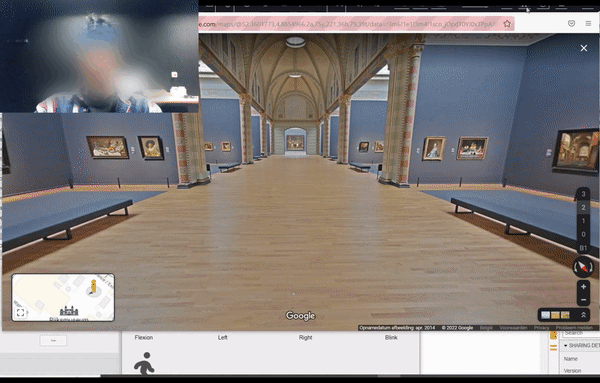

Supplement: Supplementary file 1 [file sensors-23-01704-s001.zip › Figure S1-Navigation in the Rijksmuseum.gif]

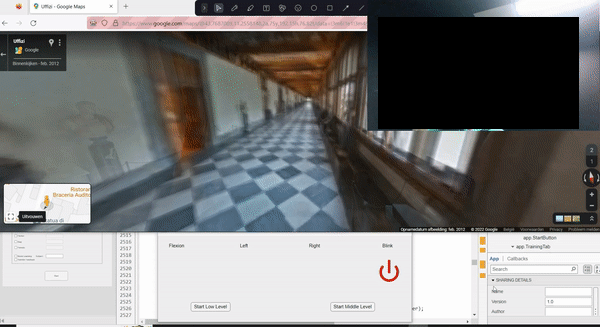

Supplement: Supplementary file 1 [file sensors-23-01704-s001.zip › Figure S2-Navigation in the Uffizi gallery.gif]

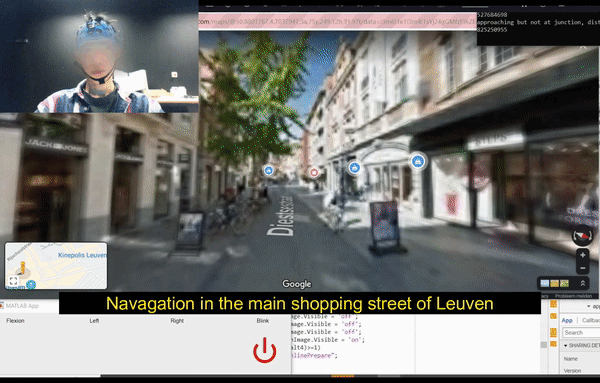

Supplement: Supplementary file 1 [file sensors-23-01704-s001.zip › Figure S3-Navigation in a city.gif]
